# The Results of 116 Operations upon the Radial Nerve Because of Lesions from War Projectiles

**Published:** 1918-01

**Authors:** 


***The Results of 116 Operations upon the Radial Nerve because of Lesions from War Projectiles***. Performed by René DUMAS : Reported by Th. TUFFIER. Trans. and Abs. from the *Bull. et Mém. de la Soc. de Chir.*, June, 5, 1917.

It has been possible for D. to watch carefully 115 cases operated by him for a sufficiently long time to establish the results of the treatment. 18 operations were for compression; 41 for incomplete section, and 56 for complete section.

In 15 of the cases of *compression* the radial nerve was confined in the callus of a fractured humerus; in 3, it was caught in the fibrous union or in scar tissue. The seat of the injuries was the trunk of the radial nerve just above the elbow, or else the radio-circumflex trunk. The operations were performed from 65 to 113 days after the injuries. In these cases the operation consisted in the careful liberation of the nerve, generous resection of the surrounding scar-tissue and the protection of the nerve by a muscular layer, a fat graft (aponeurotic or peritoneal), or some inorganic substance (sheaths of rubber or aluminum paper). Results 6 o/o of the cases did not improve ; 11 o/o improved, but regeneration is still incomplete; 83 o/o showed complete regeneration.

Of 41 cases of *incomplet section,* which were operated about 100 days after the wounds were received, 3 were for scattered perforations ; 38 were for lateral section with resulting neuroma of reaction. 37 were of the radial nerve above the elbow and 4 of the radio-circumflex trunk of the brachial plexus. The operation consisted in freeing the nerve from the neuroma, from contact with neighboring tissues, and from scar-tissues. There was no resection of the nerve itself. The same methods of protection were employed as are mentioned above. *Results:* 19 o/o no improvement; 11 o/o incomplete regeneration; and 70 o/o complete regeneration.

Of 56 cases of *complete section*, treated from three to eleven months after the wounds were received, 10 were for complete discontinuity of the two parts of the severed nerve; 46 for interposition of scar tissue between the two ends of a severed nerve which was otherwise intact. There was a considerable variety of lesions, and all occurred on the trunk of the nerve in the arm. A delay before operating, of at least 6 weeks after the cessation of suppuration, appears to the author to be essential if reinfection is to be avoided. In badly infected fractures this allowance should be doubled.

Of the 10 cases of *discontinuity* of the severed nerve, only 3 were sutured successfully, that is, without severe tension. In 3 cases the ends were brought directly together, 2 sutures were effected by doubling of the lower end and one by doubling of the upper end. In one case the two ends were brought within 3 cm. by a linen thread in the manner of Assaky. *Results :* 10 o/o incomplete regeneration ; no complete regeneration.

Of the 46 cases in which the severed nerve was interrupted by other tissues, but was otherwise intact, 4/5 offered a newly formed tissue by which the ends could be joined. The results prove the value of such natural means. The operation consisted in dissecting the bridging scar, liberating the nerve ends, and protecting the nerve from any possible pressure by means similar to those mentioned above. (The writer notes that fat should not be used unless the wound is perfectly aseptic.)

*Results* of liberation : 47 o/o showed no improvement; 10 o/o incomplete regeneration, 43 o/o complete regeneration. 10 of the 18 men returned to auxiliary service and 5 to active service.

The *total results* of the 115 operations were : regeneration in 65.2 o/o of the cases; and complete recovery in 42.6 o/o.

The writer believes that the failure in all but one of the 10 cases of suture was due to delay or to difficulty in operation.

*Le Gérant :* O. Porée. Paris — imp. Lahure, 9, rue de Fleurus.

